# Development and evaluation of a novel series of Nitroxoline-derived BET inhibitors with antitumor activity in renal cell carcinoma

**DOI:** 10.1038/s41389-018-0093-z

**Published:** 2018-11-02

**Authors:** Wei Chen, Hao Zhang, Zhifeng Chen, Hao Jiang, Liping Liao, Shijie Fan, Jing Xing, Yiqian Xie, Shijie Chen, Hong Ding, Kaixian Chen, Hualiang Jiang, Cheng Luo, Mingyue Zheng, Zhiyi Yao, Yiran Huang, Yuanyuan Zhang

**Affiliations:** 1 Department of Urology, Renji Hospital, School of Medicine, Shanghai Jiao Tong University, 160 Pujian Road, Shanghai, 200127 China; 2 Shanghai Institute of Materia Medica, Chinese Academy of Sciences, 555 Zuchongzhi Road, Shanghai, 201203 China; 3 University of Chinese Academy of Sciences, Beijing, 100049 China; 4 School of Pharmacy, China Pharmaceutical University, Nanjing, 210009 China; 5 College of Chemical and Environmental Engineering, Shanghai Institute of Technology, Shanghai, 210032 China

## Abstract

Small molecular inhibitors targeting BRD4 family proteins are emerging as promising therapies in many types of human malignancies. However, whether BRD4, as well as other BET family members, may serve as therapeutic targets in renal cell carcinoma (RCC) remains unknown. In this study, we found that both BRD2 and BRD4 were over-expressed in RCC tissues, knock-down both of which achieved potent anti-proliferative effects in RCC cells. A novel category of BET inhibitors, originated from an approved drug Nitroxoline, were synthesized and evaluated with biochemical and cellular assays, as well as the method of crystallography. The complex crystal structures of several compounds in this category with the first bromodomain of BRD4 (BRD4-BD1) were solved, revealing the binding mechanism and facilitating further structural optimizations. Among them, compound BDF-1253 showed an approximately four-fold improvement in BRD4 inhibition compared with the prototype Nitroxoline and had good selectivity for BET proteins against other bromodomain proteins or epi-enzymes in biochemical assays. Compound BDF-1253 efficiently suppressed the expression of BET downstream genes, impaired RCC cells viability via inducing cell cycle arrest and apoptosis, and decreased tumor growth in RCC xenografts. In summary, these results suggest that inhibition of BET family members has great therapeutic potentials in the treatment of RCC, and the novel series of BET inhibitors reported here are promising to become RCC drug candidates.

## Introduction

Acetylation is an important and widespread form of post-translational modification, which plays crucial roles in epigenetic regulation. Accumulating evidence has proven that epigenetic proteins could become therapeutic targets for the treatment of human malignancies and other diseases^1,2^. The bromodomains (BRDs) usually serve as a module for recognition of acetylated lysine residues. Human proteome contains 61 BRDs, which exist in 46 BRD-containing proteins^3^. The bromodomain and extra-terminal (BET) protein family has four members, including BRD2, BRD3, BRD4, and BRDT. Under normal conditions, BRD4 is involved in the regulation of transcriptions^4,5^. However, BRD4 has been found to be involved in various kinds of cancers and other diseases^6^, for its regulation of several oncogenic and antiproliferative factors, including c-Myc and Bcl-2. Emerging evidence shows that BRD4 and other BET family members could become novel therapeutic targets of cancers^7–10^. And BET inhibitors have already shown promising potentials in the treatment of several categories of cancers^11,12^. However, the efficacy of BET inhibitors in renal cell carcinoma (RCC) was poorly evaluated, and it remained to be answered whether BRD4, as well as other BET family members, can serve as therapeutic targets for the treatment of RCC.

RCC, a common genitourinary human malignancy, is usually insensitive to cytotoxic chemotherapies. The discovery and validation of novel targets are crucial for the development of new therapeutics and agents for RCC treatment. Evidence showed that c-Myc is essential for the proliferation and survival of RCC^13^. As it was revealed that BRD4 inhibition decreased the expression and protein abundance of c-Myc and related downstream genes^14–17^, we suppose inhibitors targeting BRD4 or other members in BET family might have therapeutic potentials in the treatment of RCC^18–21^. Although several series of BET inhibitors have been reported^22–24^, it is worthwhile to develop novel inhibitors with different chemical skeletons, which might have improved drug-like properties and could be used in specific clinical applications.

In this study, we showed knocking down both BRD2 and BRD4 suppressed the proliferation of RCC cells much more effectively than knocking down any single target. A novel category of BET inhibitors was synthesized and evaluated through biochemical and cellular assays. Originated from the approved drug Nitroxoline and its analogues, these compounds were more effective, and inhibited the BRD4-BD1 with satisfactory potency. The complex crystal structures of several compounds with BRD4-BD1 were solved, which revealed the binding mechanism, as well as facilitated to explain the structures and activities relationship of these inhibitors. Among them, compound BDF-1253 exhibited effective inhibition against the proliferation of RCC cell lines, as well as tumor growth on the xenograft mice model. BDF-1253 selectivity inhibited all BET proteins with minimal effect on the other BRD-containing proteins or epigenetic enzymes, thus it is a selective and potent BET inhibitor. This novel series of BET inhibitors is promising to become drug candidates after further optimization, for the treatment of RCC.

## Results

### The role of BET family members in renal cell carcinoma

**Fig. 1:**
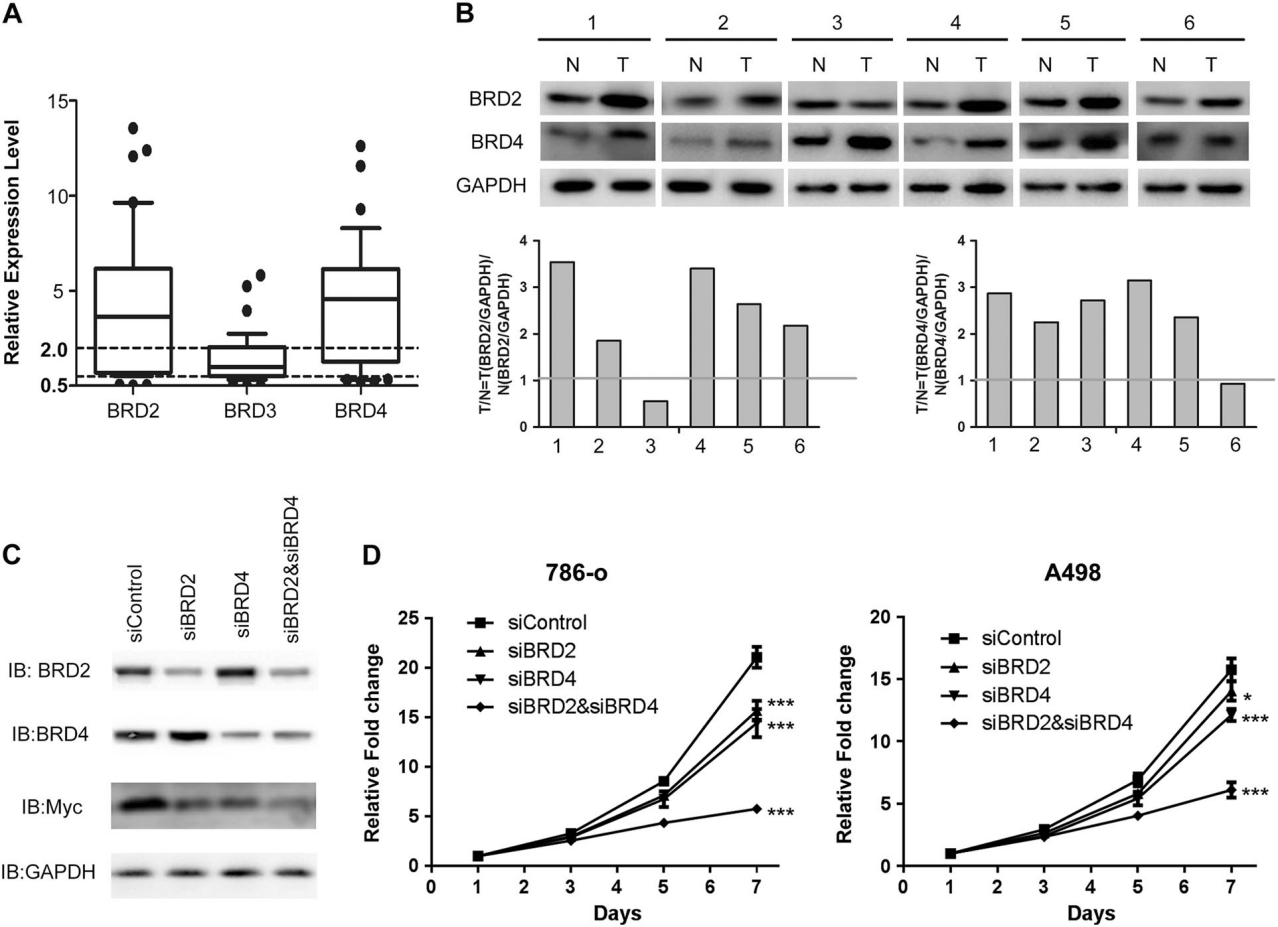
Overexpression and knockdown of BET proteins in RCC. **a** Quantitative real-time PCR results of relative expression levels of BRD2, BRD3, BRD4 in 39 pairs of RCC tissues and adjacent normal tissues. **b** Immunoblot analysis of BRD2 and BRD4 expression in 6 pairs of RCC tissues and adjacent normal tissues. Quantifications are shown in the panels below. **c** Knockdown efficiency of siBRD2 and BRD4 by immunoblot analysis. **d** Knockdown of BRD2 or BRD4 moderately inhibited the proliferation of RCC 786-O and A498 cells, while knocking down both of their expressions resulted in more significant anti-proliferative effects. Two-way ANOVA with multiple comparisons was used to compare each knocking down group to siControl group. Representative results were shown, error bars indicated standard deviation among technical replicates (*n* = 5 technical replicates per experiment). *p < 0.05; **p < 0.01; ***p < 0.001. The experiments were replicated 3 times to confirm the conclusion

To examine whether BRD4, as well as other BET family members, may serve as potential therapeutic targets in RCC, we first investigated their roles in RCC. Using real-time RT-PCR, the relative expression levels of BRD2, BRD3, and BRD4 were evaluated by the comparison of their expression in 39 pairs of RCC tissues and adjacent normal tissues. It was found that both BRD2 and BRD4 were over-expressed in RCC tissues (Fig. 1a). The upregulation of BRD2 and BRD4 was further confirmed in paired RCC tissues by immunoblotting (Fig. 1b). Knocking down both BRD2 and BRD4 significantly reduced expression of oncogenic c-Myc, one of the downstream effectors of BET proteins (Fig. 1c). Moreover, knocking down of BRD2 or BRD4 moderately inhibited the proliferation of RCC 786-O and A498 cells, while knocking down both of them resulted in more significant anti-proliferative effects (Fig. 1d). These findings suggested that BET proteins played important roles in RCC and might be potential targets for the treatment of RCC. To test whether targeting BET family members is effective for RCC treatment, we next developed novel BET inhibitors and evaluated their anti-proliferative and anti-tumor effects in RCC cells and mice model.

### Development of novel BET inhibitors

**Fig. 2:**
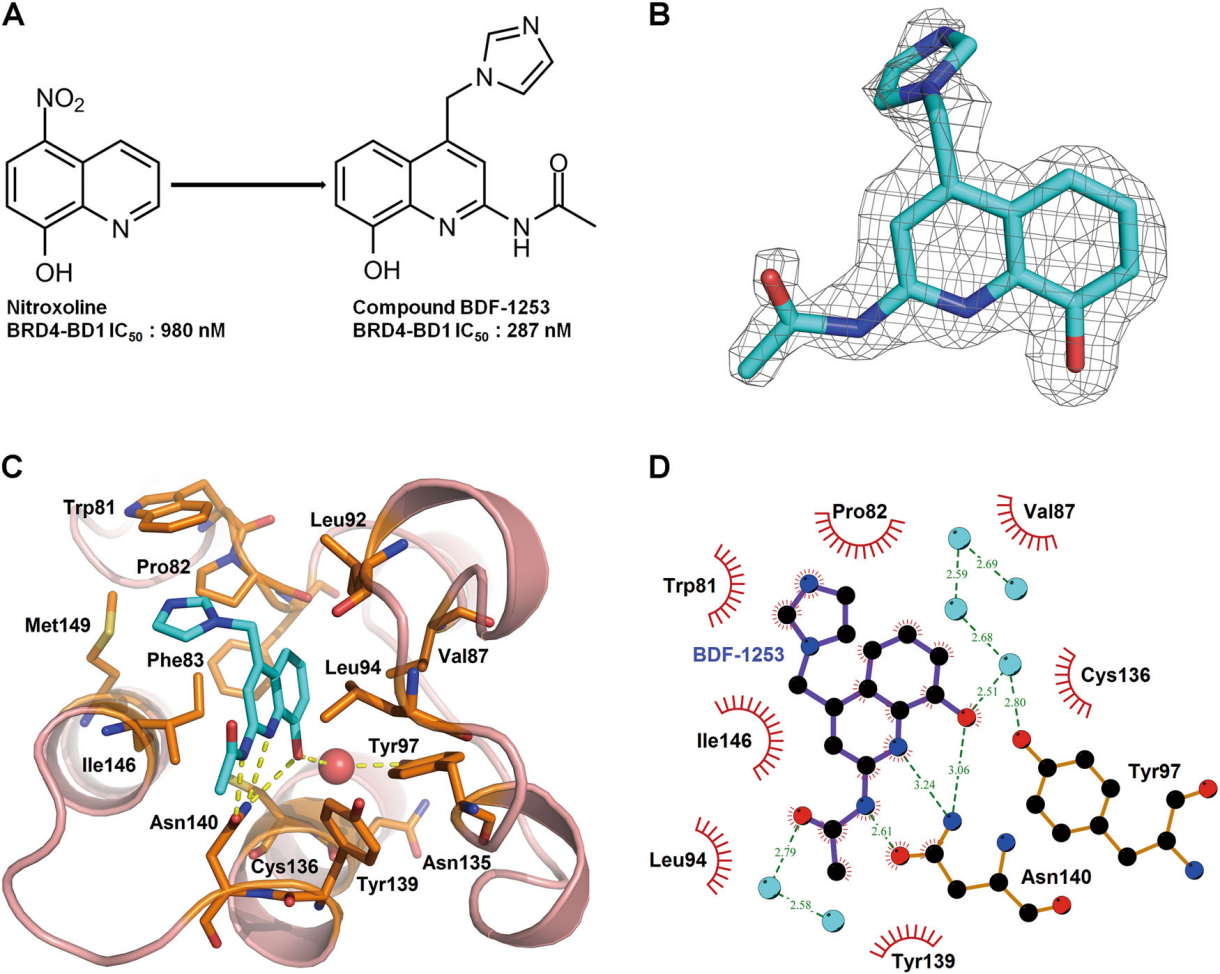
Complex crystal structure of BRD4-BD1 with compound BDF-1253. **a** The modification from Nitroxoline to compound BDF-1253 increased potency against the BRD4-BD1. **b** The 2Fo–Fc electron density map of compound BDF-1253, in its complex crystal structure with BRD4-BD1 (PDB code: 5Z5V). The density contour level was set to 1.0 sigma. **c** Compound BDF-1253 within the binding pocket of BRD4-BD1, direct and water-bridged hydrogen bonds were formed between the compound and surrounding residues. **d** Hydrophobic contacts of compound BDF-1253 with surrounding residues within the binding pocket

We previously reported Nitroxoline and its analogues as effective BRD4-BD1 inhibitors^25^. Based on these findings, we synthesized a novel series of BRD4-BD1 inhibitors, most of which showed significantly improved potency (Fig. 2a). These novel inhibitors, with their names, structures, and inhibitory potency against BRD4-BD1 binding with acetylated lysine, are listed in Table S1 (refer to supplementary methods for synthesis, purification, and characterization of these compounds).

In terms of structure and activity relationship, compound BDF-2254 had improved potency in comparison with BDF-2141, indicating that the introduction of a hydroxyl group was favorable. And obvious potency improvement was observed for compound BDF-2265, after the substitution of amine group on the 8-hydroxyquinoline into the –NH–CO–CH_3_ group. Similar situations were observed for compound BDF-2143 and BDF-2243. Generally, the introduction of a methylene group between the smaller ring and the 8-hydroxyquinoline ring increased the potency. Introduction of the third nitrogen atom into the five-membered ring decreased the potency. This situation was applicable to the compounds BDF-2030, BDF-2268, BDF-2271, the compounds BDF-1031, BDF-1253, BDF-1252, and the compounds BDF-2242, BDF-2260, BDF-2261.

### Complex crystal structures of BRD4-BD1 with BDF-1253, and BDF-2141, BDF-2254

To reveal the binding mechanism and facilitate further optimization, the complex crystal structures of several compounds in this category with BRD4-BD1 were determined. The crystal structure of compound BDF-1253 in complex with BRD4-BD1 was solved at the resolution 1.66 Å, with PDB code 5Z5V. While the complex crystal structure of compound BDF-2141 was solved at the resolution 1.99 Å, with PDB code 5Z5T. And the crystal structure of compound BDF-2254 in complex with BRD4-BD1 was solved at the resolution 1.63 Å, with PDB code 5Z5U. The electron density maps of compounds in the solved crystal structures were intact (Fig. 2b and Supplementary Figure S1).

The binding modes of these compounds in the pocket were almost the same, in terms of their shared part. These compounds formed direct hydrogen bonds as well as water-bridged hydrogen bonds (Fig. 2c and Figure S1). These compounds formed multiple hydrogen bonds with the residue Asn-142. Similar to the prototype Nitroxoline, a water-bridged hydrogen bond was formed between compound BDF-1253 and the residue Tyr-97. In addition, hydrophobic contacts were formed with the surrounding hydrophobic residues, including Trp-81, Pro-82, Phe-83, and Val-87, Leu-92, Leu-94, Ile-146 (Fig. 2d).

Compared to compound BDF-2141, compound BDF-2254 had improved potency due to the introduction of a hydroxyl group. In the complex crystal structure of compound BDF-2254 with BRD4-BD1, a hydrogen bond was formed between this hydroxyl group and the backbone carbonyl group of the residue Pro-82. In addition, two water-bridged hydrogen bonds were formed between this hydroxyl group and the crystal waters within the binding pocket, also stabilizing the binding between compound BDF-2254 and the pocket. Taken together, the solved crystal structures showed that these inhibitors have the same binding mode, which was consistent with their structure and activities relationship. Multiple hydrogen bonds were formed between the compounds and the single residue Asn-142, which could explain the relatively higher potency of these novel BRD4-BD1 inhibitors, compared with the prototype Nitroxoline.

We chose one of the most potent inhibitors, BDF-1253, for further selectivity analysis and functional study. BDF-1253 showed four-fold improvement in BRD4 inhibition in vitro. First, we compared the inhibitory activity of BDF-1253 to displace BRD4-BD1 or BRD4-BD2 domain from acetylated H4 peptide using ALPHA assay. As shown in Supplementary Figure S2A, BDF-1253 showed no obvious selectivity for BRD4-BD1 or BRD4-BD2 domain. An in vitro selective assay was also conducted to examine the effect of BDF-1253 at 3 μM on the other BET proteins, other BRD proteins out of BET family (SMACAR, BPTF, PCAF) as well as several epigenetic enzymes. As shown in Supplementary Figure S2B, BDF-1253 selectively inhibited BET family proteins with minimal effects on non-BET BRD-containing proteins or other epigenetic enzymes, based on which it should be defined as a selective BET inhibitor.

### Compound BDF-1253 inhibited the proliferation of RCC cells in vitro and tumor growth in vivo

**Fig. 3:**
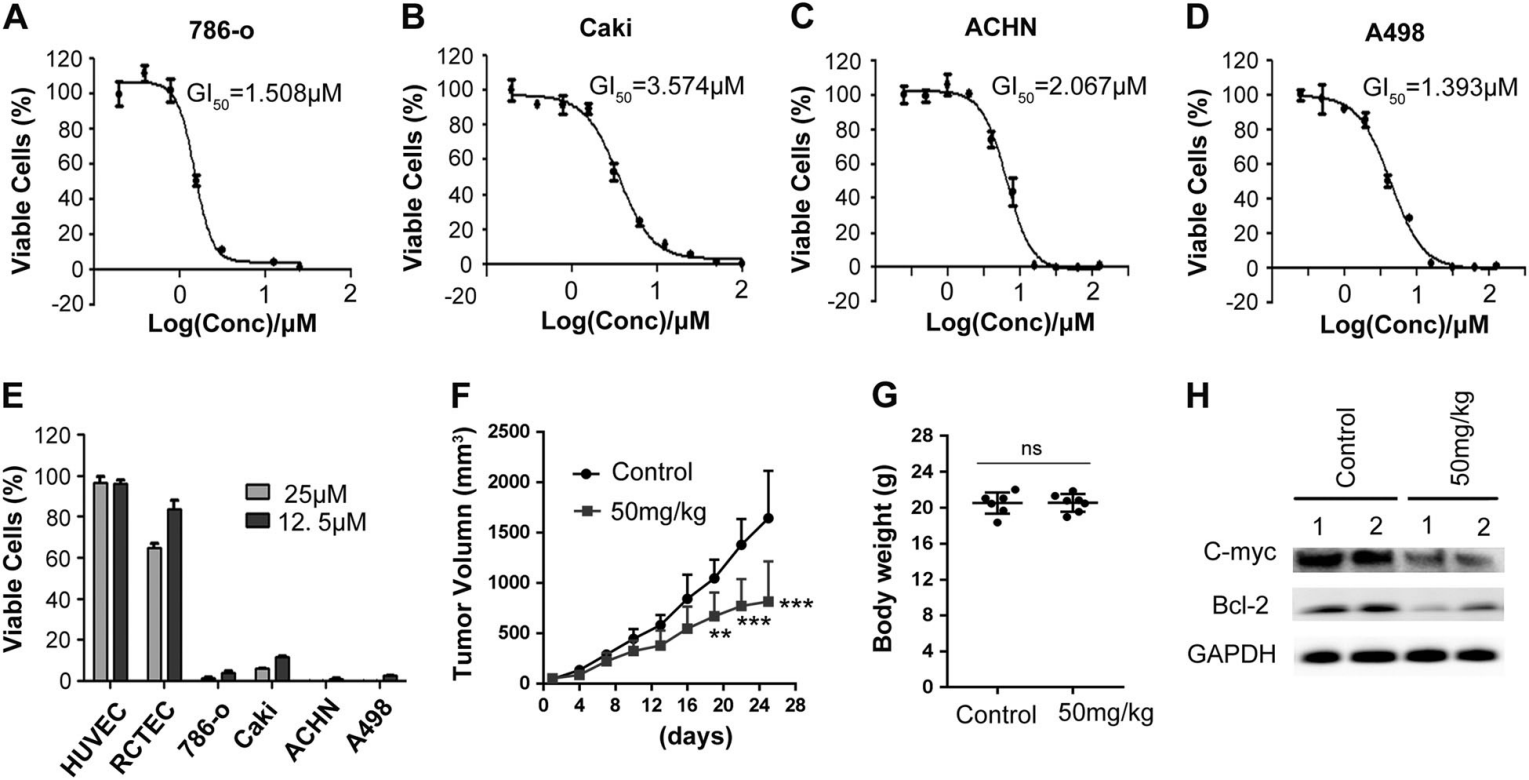
Compound BDF-1253 effectively inhibited the proliferation of RCC cell lines. **a** Compound BDF-1253 inhibited the proliferation of RCC 786-O cells, with an IC_50_ value of 1.508 μM for the treatment of 7 days. **b** Compound BDF-1253 inhibited the proliferation of RCC Caki cells, with an IC_50_ value of 3.574 μM for the treatment of 7 days. **c** Compound BDF-1253 inhibited the proliferation of RCC ACHN cells, with an IC_50_ value of 2.067 μM for the treatment of 7 days. **d** Compound BDF-1253 inhibited the proliferation of RCC A498 cells, with an IC_50_ value of 1.393 μM for the treatment of 7 days. **e** Compound BDF-1253 effectively inhibited the proliferation of four RCC cell lines, without obvious cytotoxic effects against normal cells. Data were represented relative to DMSO treatment. **a**–**e** Error bars indicate standard deviation among 3 biological replicates. **f** Significant reduction in tumor size was observed after the treatment of compound BDF-1253 for 21 days, on RCC xenograft mice model. Two-way ANOVA with multiple comparisons was used for comparison. Error bars indicated standard deviation (*n* = 7 mice in each group). *p < 0.05; **p < 0.01; ***p < 0.001. **g** Mice body weight was not obviously affected after compound treatment. Statistical significance relative to the control group was assessed using one-way ANOVA. ns indicated not significant. **h** Compound treatment reduced expression of BET downstream effector c-Myc and Bcl-2 in vivo

We further assessed the efficacy of compound BDF-1253 (Fig. 2a) in a panel of RCC cells. Four RCC cell lines were treated with compound BDF-1253 of different concentrations and subjected to Alamar Blue Assay. Then viability percentages were normalized and the IC_50_ values were fitted. The IC_50_ value of compound BDF-1253 treatment for 7 days were 1.508, 3.574, 2.067, and 1.393 μM, for the 786-O, Caki, ACHN, and A498 cell lines, respectively (Fig. 3a–d). Treatment of compound BDF-1253 effectively suppressed the cell viability of RCC cells, with little inhibition on HUVEC and RCTEC normal cell lines at similar concentrations (Fig. 3e). We next determined the antitumor effects of compound BDF-1253 in RCC xenograft mice model. A remarkable reduction in tumor size was observed after the treatment with compound BDF-1253 for 21 days (Fig. 3f). In terms of toxicity, no obvious loss of body weight or toxicity was observed during the drug dosing of compound BDF-1253 (Fig. 3g). Immunoblotting analysis of tumors revealed c-Myc expression is reduced after compound treatment, suggesting effective inhibition of BET proteins in vivo (Fig. 3h). These data revealed that inhibition of BET functions could inhibit RCC cell proliferation and tumor growth, suggesting that BET proteins may serve as anti-tumor targets in RCC.

### Compound BDF-1253 induced G0/G1 cell cycle arrest and apoptosis in RCC cell lines

**Fig. 4:**
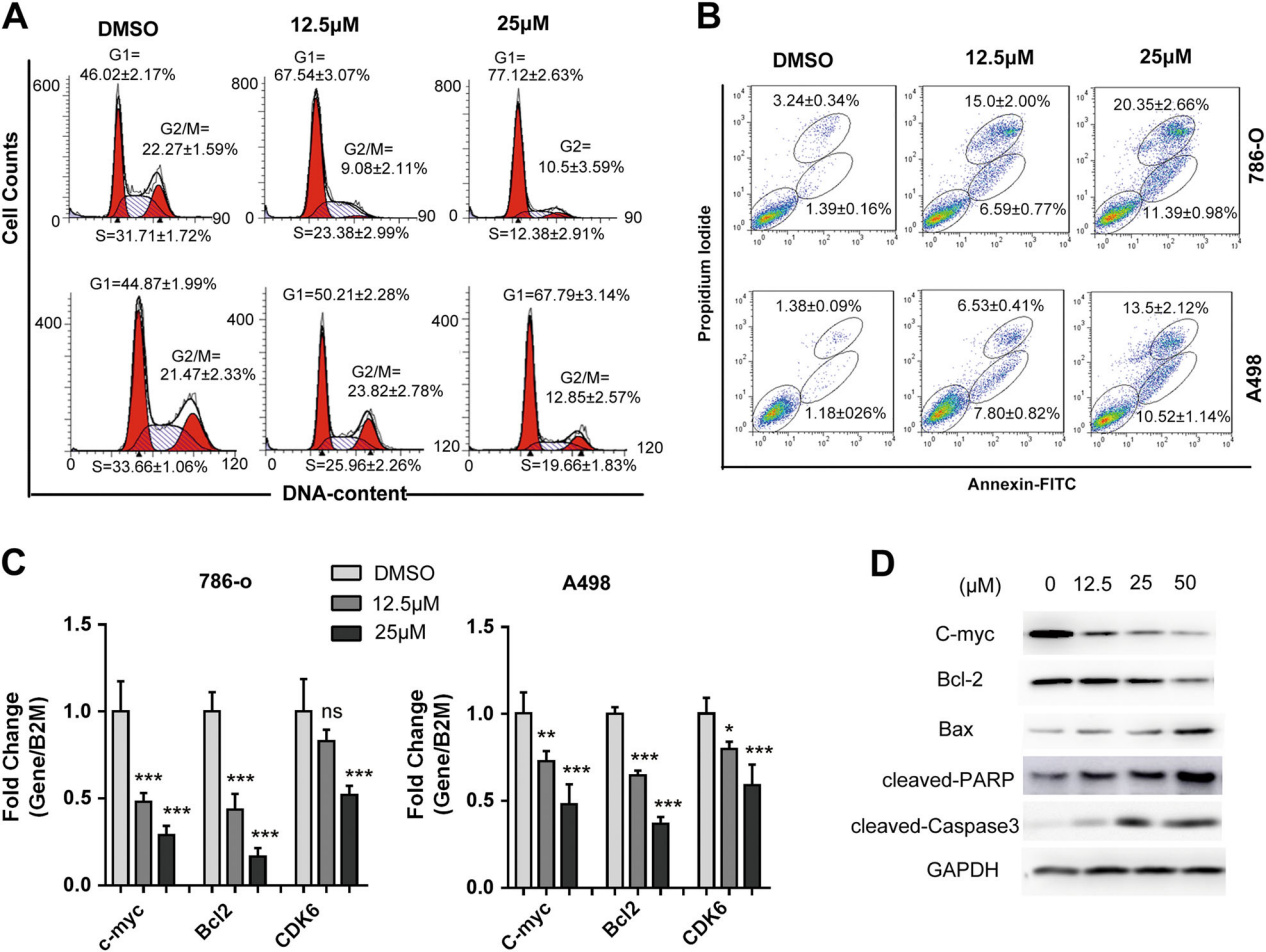
Cell cycle arrest and apoptosis induced by compound BDF-1253. **a** Compound BDF-1253 induced cell cycle arrest at G0/G1 phase in RCC 786-O and A498 cells, compared to the untreated control. Data was shown in the figure as mean ± SD. Experiments were performed in *n* = 3 biological replicates. **b** Compound BDF-1253 induced apoptosis in RCC 786-O and A498 cells, in a dose-dependent manner. Representative results were shown, data was shown in figure as mean ± SD. Experiments were performed in *n* = 3 biological replicates. **c** Compound BDF-1253 decreased the mRNA expression levels of c-Myc, Bcl-2, and CDK6, in a dose-dependent manner. Gene-specific data were normalized to β2-microglobulin expression and shown as average expression relative to DMSO control. Standard deviations among 3 biological replicates were indicated as error bars. One-way ANOVA was used to compare compound treatment group to DMSO control. *p < 0.05; **p < 0.01; ***p < 0.001. **d** Compound BDF-1253 decreased the protein abundance of c-Myc, Bcl-2, and increased the protein abundance of Bax, cleaved-PARP, cleaved-Caspase3 in a dose-dependent manner

To reveal the anti-proliferative mechanism of these BET inhibitors, we assessed the effect of compounds treatment on cell cycle progression and apoptosis. 786-O and A498 cells were treated with the compound BDF-1253, and subjected to flow cytometry analysis. Compound BDF-1253 induced cell cycle arrest at G0/G1 phase in RCC 786-O and A498 cells, compared to the untreated control (Fig. 4a). The treatment of compound BDF-1253 also induced cell apoptosis in a dose-dependent manner (Fig. 4b).

### Compound BDF-1253 repressed c-Myc expression and activate pro-apoptosis pathways

To confirm that the anti-tumor effect of compound BDF-1253 was mediated through the inhibition of BRD4, the mRNA expression and protein abundance of BRD4 downstream effectors associated with cancer development, including c-Myc, Bcl-2, and CDK6, were checked after the treatment of compound BDF-1253. The mRNA expression levels of c-Myc, Bcl-2, and CDK6 were dose-dependently decreased after the treatment of compound BDF-1253 (Fig. 4c). Consistently, the protein abundance of these genes was also found to be reduced after the treatment, in a dose-dependent manner (Fig. 4d). A negative compound BDF-1251, structurally similar but less active, had no obvious effects on RCC cell proliferation or transcription of BET target gene, suggesting the anti-proliferative effect of BDF-1251 can be mainly due to the inhibition on BET proteins (shown in Supplementary Figure 3). Moreover, the treatment of compound BDF-1253 resulted in increased PRAP and Caspase3 cleavage and elevated expression of pro-apoptotic protein Bax, indicating a marked induction of pro-apoptosis pathway.

**Fig. 5:**
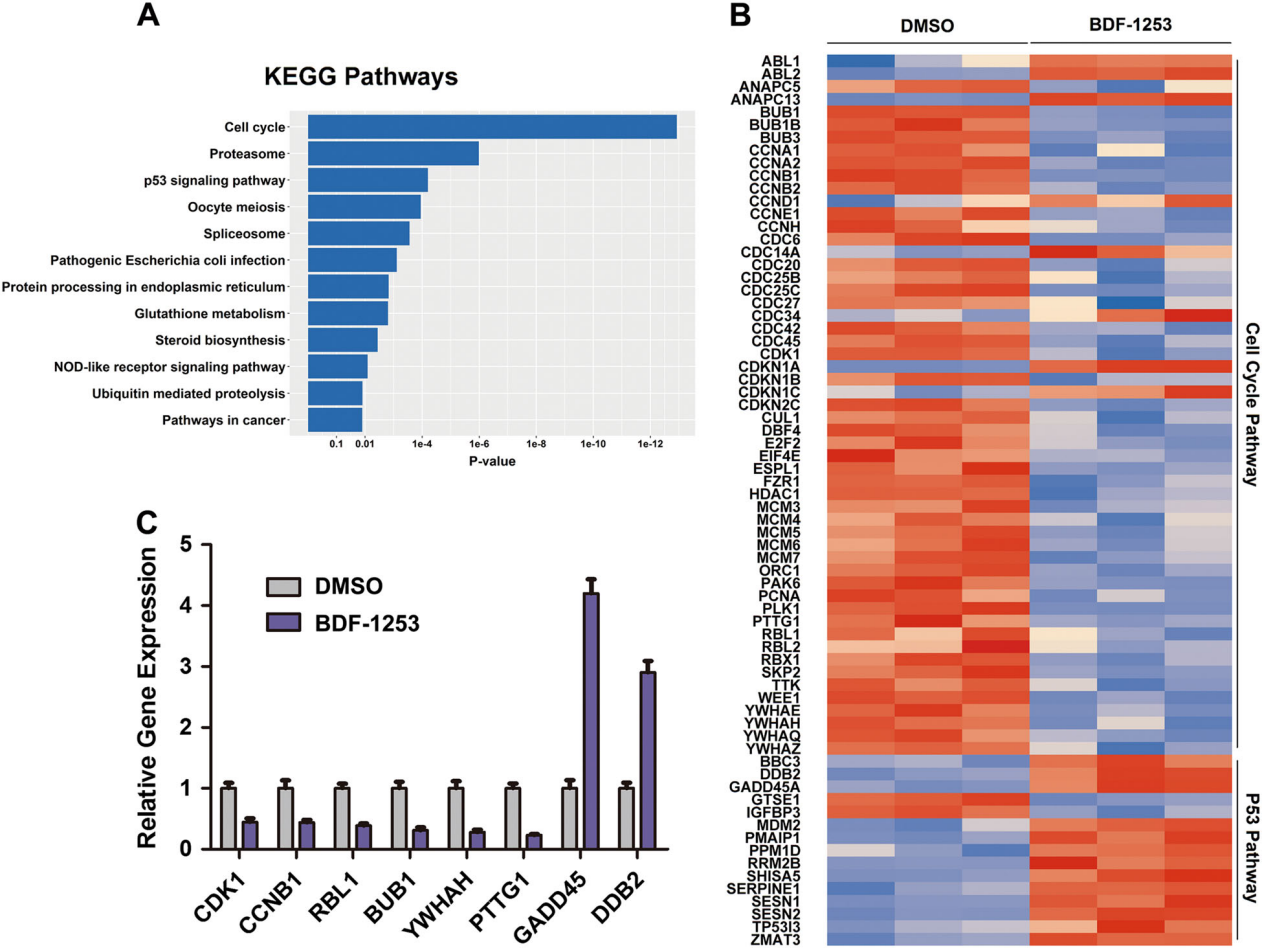
RNA-Seq data analysis and KEGG pathways statistics. **a** The *p*-values and names of the most over-represented KEGG pathways, calculated on the basis of all the differentially expressed genes after the treatment of compound BDF-1253. **b** Heatmap of all the differentially expressed genes from the cell cycle and p53 KEGG pathways, after the treatment of compound BDF-1253. **c** RT-PCR analysis validated the differentially expressed genes. Gene-specific data were normalized to β2-microglobulin expression and shown as average expression relative to DMSO control. Error bars indicated standard deviations among 3 technical replicates per experiment

To characterize the global transcriptional effects and change of signaling pathways induced by the compounds treatment, RNA sequencing was carried out in RCC 786-O cells treated with compound BDF-1253 or DMSO. Analysis of the results showed that dozens of downstream genes of BET proteins (e.g., YWHAH, PTTG1) were differentially expressed after the treatment of compound BDF-1253, in comparison with the DMSO control. In terms of the pathway over-representation analysis, the full list of over-represented KEGG pathways was summarized (*p*-value cutoff < 0.1, Fig. 5a and Supplementary Table S2), including the cell cycle and p53 pathways, which were further confirmed by RT-PCR analysis (Fig. 5c). Most of the differentially expressed genes from the cell cycle pathway were down-regulated (Fig. 5b), which was consistent with the phenotypic consequences of compound treatment.

## Discussion

As RCC is aggressive and insensitive to common chemotherapy, it is beneficial to explore new therapeutic targets. The strategy of BET inhibition has shown promising potentials in the treatment of several kinds of cancers. However, whether BET family members may serve as therapeutic targets in RCC remains poorly studied. Although the over-expression of BRD4 was reported in RCC^26^, the expressions of other BET proteins still require systematic assessment. In this study, we reported the over-expression of BET family proteins BRD2 and BRD4 in RCC. Knocking down BRD2 or BRD4 only moderately inhibited RCC cell proliferation, which might be due to the compensatory effect by other BET family members, while knocking down both BRD2 and BRD4 resulted in remarkable suppression of RCC cell proliferation and reduced expression of c-Myc onco-protein. Two previous studies^26,27^ used genetic knockdown to assess the role of BRD4 in RCC, however, the role of other members (BRD2, BRD3) in BET family in RCC remains unclear to date. Thus our study consisted of and further characterized the role of BET family members in RCC, suggesting that BET family proteins might represent potential therapeutic targets in the treatment of RCC.

Since the BET family proteins are promising drug targets, there is a great demand for the development of novel chemotypes of BET inhibitors. In our previous study, we used the strategy of drug repurposing to find BET inhibitors, and reported the antibiotic Nitroxoline as BET inhibitor. On the basis of these findings, a novel series of BET inhibitor were synthesized and evaluated. Derived from the prototype Nitroxoline, these inhibitors showed improved potency. They competitively bound the first bromodomain of BRD4, thus inhibited its binding with acetylated H4 peptide, with similar inhibition against other BET family members.

The anti-tumor efficacy of compound BDF-1253 was assessed in RCC cells and mice model. Compound BDF-1253 effectively inhibited the proliferation of RCC cell lines without obvious inhibition against normal cell proliferation, suggesting its safety when applied in vivo. It also attenuated tumor growth in RCC xenograft mice model as a single agent without obvious toxicity, suggesting its translational potential in the treatment of RCC. In terms of the underlying mechanism, it was revealed that the anti-proliferative effect of compound BDF-1253, is via the induction of cell cycle arrest and apoptosis. In addition, compound BDF-1253 suppressed the expression of BRD4 downstream genes c-Myc and Bcl-2, as well as increased PRAP and Caspase3 cleavage and pro-apoptotic protein Bax expression. Consistent with these results, the RNA-Seq data analysis indicated that the efficacy was mainly mediated through the differential expression of dozens of genes from the cell cycle and p53 KEGG pathways.

Taken together, a novel category of BET inhibitors was reported and their anti-tumor effects were evaluated in RCC. The solved complex crystal structures revealed a binding mechanism, and facilitated further optimization and development. Moreover, the results in this study established proof of concept for targeting BET family proteins with these novel inhibitors in the treatment of RCC and investigated into its underlying mechanism.

## Materials and methods

### Patients and tissue samples

Human samples were conducted in Renji Hospital of Shanghai Jiaotong University. The study protocol is approved by institutional ethics committee of Renji Hospital and strictly followed institutional ethical board guidelines. Informed consent forms were signed from the patients prior to participation in the study. 39 patient ccRCC tissues and paired non-tumorous tissue samples were collected from patients at the time of surgical removal of tumor tissues and stored in liquid nitrogen. The pathologic diagnosis of ccRCC was determined by two pathologists separately.

### Real-time PCR analysis

Trizol reagent (Vazyme) was used to extract total RNA of ccRCC tumor samples or cells following manufacturer’s instructions. Synthesis of complementary DNA (cDNA) was done using the HiScript® II RT SuperMix (Vazyme). StepOnePlus (Life Technologies) apparatus was applied to carry out quantitative real-time PCR (qPCR). The primers are listed in Table S3. Gene-specific data were normalized to β2-microglobulin and are calculated as relative expression compared to DMSO or control group using the ΔΔCT method.

### Western blot

Protein lysates from tissue samples were obtained using RIPA buffer supplemented with protease and phosphatase inhibitors. For the cellular samples, cells were collected following treatment with DMSO and compounds. Western blot was performed as previously described.^25^ The primary antibodies were BRD2 (A302-583A, Bethyl), BRD3 (A302-368A, Bethyl), BRD4 (A301-985A, Bethyl), MYC (sc-40, Santa Cruz Biotechnology), BCL2 (ab7973, Abcam), BAX (ab32503, Abcam), cleaved PARP (Cell Signaling, #5625). The secondary antibodies were anti-rabbit IgG (Jackson, 111-035-003) or anti-mouse IgG (Jackson). HRP substrate (GE Healthcare) was used for the detection of chemical luminescence, and FUJIFILM LAS-4000 was used for image acquisition.

### Knock down

siRNAs for human BRD2, BRD3, and BRD4 as well as control siRNA were purchased from Genepharma Inc. Cells were transfected with siRNA following the manufacturer’s protocols. The target sequences of the siRNA are listed as follows:

siBRD2: 5′-CCGGAAGCCCUACACCAUUAA-3′;

siBRD3: 5′-GCTGAUGUUCUCGAAUUGCUA-3′;

siBRD4: 5′-GAACCUCCCUGAUUACUAU-3′;

nonspecific control siRNA: 5′-ACAGACUUCGGAGUACCUG-3′.

### Protein expression and purification

Proteins expression and purification were done as previously described^22^. pET28a-BD4-BD1 plasmid was transfected into *Escherichia coli* BL21 (DE3) cells for expression of BD4-BD1 with an N-terminal 6× histidine tag. Cultures were grown to an OD600 value of 0.6–0.8 at 37 °C and inducted at 16 °C in the presence of 0.4 mM IPTG. Cells were lysed in lysis buffer (20 mM HEPES, pH 7.4, 150 mM NaCl, 10 mM imidazole, 0.1% β-mercaptoethanol) and supernatant was loaded on to nickel affinity chromatography (HisTrap FF, GE Healthcare). The proteins were eluted with elution buffer (20 mM HEPES, pH 7.4, 150 mM NaCl, 350 mM imidazole, 0.1% β-mercaptoethanol) and further purified by gel-filtration chromatography (Superdex 75, GE Healthcare). Purified proteins were concentrated in storage buffer (20 mM HEPES (pH 7.4), 150 mM NaCl, and 1 mM dithiothreitol (DTT)). For subsequent assays, his-tag was removed by treating with TEV protease.

### ALPHA screen assay

Amplified Luminescent Proximity Homogeneous Assay (ALPHA) screen assay was used to determine the IC50 of compounds for competitive displacement of BRD4_BD1 from the acetylated H4 peptide. As previously described^25^, 2.5 μL compounds and 2.5 μL proteins were added into each well of 384-well plates and incubated at room temperature for 15 min. To start the reaction, 5 μL substrate peptide was added and incubated at room temperature for 5 min. Subsequently, 5 µL nickel-chelate acceptor beads (PerkinElmer) and 5 µL streptavidin-conjugate donor beads (PerkinElmer) were added. After 60 min incubation at room temperature, data were collected by EnVision (PerkinElmer) and analyzed using GraphPad Prism 5.0.

### Complex crystal structure determination

Crystals of BRD4-BD1 were obtained using the method of sitting drop vapor diffusion. After testing different pool conditions, pool condition of 10–15% glycerol and 4 M sodium formate was chosen to obtain diffraction-quality protein crystals. Then protein crystals were soaked into the reservoir solution containing various concentrations of compounds. The X-Ray diffraction data was obtained using BL17B1 and BL19U beamlines of Shanghai Synchrotron Radiation Facility (SSRF), at 100 K. Then diffraction data was processed and integrated using the software XDS^28^. After data scaling using the Aimless module of the CCP4 software package^29^, the structure was solved using the Phaser module^30^ of the Phenix software package^31^, via the method of molecular replacement using the template of PDB code 2OSS. LigandFit module of Phenix^32^ and Coot^33^ were used to fit the ligands into the electron density map. The final complex structure was obtained after rounds of refinement and adjustment using Phenix and Coot. After structure validation using MolProbity^34^, the final complex structures of BRD4-BD1 with compounds were deposited to the RCSB Protein Data Bank. Summary of data statistics is presented in Supplementary Table S1.

### Cell culture and viability assay

RCC cells 786-O, 769-P, A498, and ACHN were purchased from ATCC. All the cells were authenticated by STR profiling at Genesky Biopharma Technology and tested for Mycoplasma contamination. Cells were cultured in a culture medium suggested by ATCC and incubated at 37 °C with 5% CO_2_. For the viability assay, cells were plated on 96-well plates at the density of 0.5–1 × 10^3^/well. After cell attachment, various concentrations of compounds and DMSO were added, the plates were incubated for 7 days. After the addition of 10 μL Alamar Blue and incubation for 3 h at 37 °C, fluorescent signals were measured with a PE Envision reader, at the wavelength of 544 nm for excitation and 590 nm for emission. Finally, the percentages of viability were calculated by normalizing against the control^35^.

### Flow cytometry

For the cell cycle assay, cells were grown on 6-well plates and treated with compound BDF-1253 of different concentrations and DMSO for 48 h. Cells were trypsinized and collected by centrifugation. After washing with PBS, pre-cooled 70% ethanol was added to the sample for fixation overnight. Fixed cells were collected, washed with PBS, and then resuspended at the density of 1 × 10^6^ in PI/RNase solution for the cell cycle analysis using BD Flow Cytometry. For apoptosis analysis, cells were resuspended in 300 μL binding buffer and stained using the Annexin V-FITC/PI Apoptosis Detection Kit (Vazyme). Data were collected and analyzed using BD Flow Cytometry.

### RNA-Seq data analysis and pathways statistics

To characterize the genomic impact of these inhibitors, 786-O cells were treated with DMSO and compound BDF-1253 of 12.5 and 25 μM. After 24 h, total RNA was isolated and purified using DNaseI (Takara) and Dynabeads Oligo (dT)25 (Life Technologies). Then purified RNA of 100 ng was used for cDNA library construction, using the NEBNextUltra^TM^ RNA Library Prep Kit for Illumina (NEB). Sequencing data was collected on an Illumina HiSeq 2500 instrument, and paired-end reads were processed using the Tophat2 software package^36^, with the GRCh38/hg18 Ensembl transcript set. The Cufflinks software package^37^ was used to assemble transcripts, and the transcriptome for all the samples was assembled using Cuffmerge. Finally, differentially expressed genes were identified using Cuffdiff. The Kyoto Encyclopedia of Genes and Genomes (KEGG) database was used for the pathways statistics. Using the GeneAnswers package^38^ of the Bioconductor project, the *p*-values of involved KEGG pathways were calculated, on the basis of all the differentially expressed genes. The heatmap and pathways histogram were plotted using the ggplot2 package of R. The raw data of RNA-Seq and processed expression files have been deposited to the Gene Expression Omnibus (GEO) under accession GSE109870.

### Tumor xenografts and in vivo treatment

All animal experiments were approved by IACUC (Institutional Animal Care and Use Committee) of Shanghai Institute of Materia Medica. Four to six-week-old female nude mice purchased from Silaike Laboratory Animal Ltd. were subcutaneously inoculated with 5 × 10^6^ 786-O cells in 100 μL PBS. All nude mice were raised under pathogen-free conditions to achieve tumor growth. Sample size of each group (*n* = 7) was chosen following the instruction by http://www.biomath.info. As long as the tumor sizes reached palpable stage, the mice were randomly divided into two groups (*n* = 7/group). One group is for compound BDF-1253 treatment trials and the other group is the control group. Investigator is blind to the reagent given to mice and did not know which group is drug or solution. These mice were treated intraperitoneally with either diluted compound BDF-1253 of 50 mg/kg or vehicle on a daily basis. Tumor volume was evaluated every 3 days using Vernier calipers and calculated with the equation: length × width^2^ × 0.5. Animals were killed when their tumors reached 2 cm^3^.

### Statistics

Data were presented as the mean ± SD. Differences between experimental groups were analyzed using one-way ANOVA or two-way ANOVA. The variance is similar between the groups that are being statistically compared. *p* *<* 0.05 was considered significant. *p *<* 0.05, **p *<* 0.01, ***p *<* 0.001. All statistical analyses were performed using GraphPad Prism software or SPSS.

### Accession numbers

The complex crystal structures of compound BDF-1253, BDF-2141, and BDF-2254 with BRD4-BD1 have been deposited to the RCSB Protein Data Bank, with the accession numbers 5Z5V, 5Z5T, and 5Z5U, respectively.

The raw data of RNA-Seq and processed expression files have been deposited to the Gene Expression Omnibus (GEO) under accession GSE109870.

## Electronic supplementary material

supplementary tables and figures
