# Supplementary material for: Development and evaluation of a novel series of Nitroxoline-derived BET inhibitors with antitumor activity in renal cell carcinoma
Source: Oncogenesis. 2018 Nov 2;7(11):83. doi: 10.1038/s41389-018-0093-z (PMC6212493; doi:10.1038/s41389-018-0093-z)
Supplement: Supplementary file 1 — supplementary tables and figures [file 41389_2018_93_MOESM1_ESM.docx]

**Supplementary tables:**

Table S1. X-Ray data collection and processing statistics.

| DataSet | BDF-1253 | BDF-2141 | BDF-2254 |
| --- | --- | --- | --- |
| PDB code | 5Z5V | 5Z5T | 5Z5U |
| Wavelength | 0.9785 | 0.9785 | 0.9785 |
| Resolution range | 40.59 - 1.66  (1.719 - 1.66) | 40.48 - 1.991  (2.062 - 1.991) | 40.54 - 1.631  (1.689 - 1.631) |
| Space group | P 21 21 21 | P 21 21 21 | P 21 21 21 |
| Unit cell | 32.037 47.293 79.081  90 90 90 | 33.099 47.337 78.122  90 90 90 | 32.006 47.276 78.794  90 90 90 |
| Total reflections | 28776 (2810) | 17727 (1737) | 30989 (3062) |
| Unique reflections | 14409 (1407) | 8867 (868) | 15497 (1531) |
| Multiplicity | 2.0 (2.0) | 2.0 (2.0) | 2.0 (2.0) |
| Completeness | 0.97 (0.97) | 1.00 (1.00) | 1.00 (1.00) |
| Mean I/sigma(I) | 23.88 (4.82) | 25.24 (6.04) | 21.69 (3.86) |
| Wilson B-factor | 15.89 | 24.12 | 16.81 |
| R-merge | 0.01724 (0.1593) | 0.01544 (0.108) | 0.01763 (0.1813) |
| R-meas | 0.02438 (0.2253) | 0.02183 (0.1527) | 0.02493 (0.2563) |
| CC1/2 | 1 (0.956) | 1 (0.989) | 1 (0.959) |
| CC* | 1 (0.989) | 1 (0.997) | 1 (0.989) |
| Reflections used in refinement | 14403 (1406) | 8848 (866) | 15487 (1527) |
| Reflections used for R-free | 699 (68) | 547 (53) | 693 (69) |
| R-work | 0.1833 (0.2007) | 0.2100 (0.2858) | 0.1939 (0.2484) |
| R-free | 0.2208 (0.2097) | 0.2509 (0.2833) | 0.2230 (0.3131) |
| CC(work) | 0.960 (0.940) | 0.961 (0.932) | 0.959 (0.916) |
| CC(free) | 0.946 (0.925) | 0.941 (0.830) | 0.941 (0.865) |
| Number of non-hydrogen atoms | 1202 | 1135 | 1203 |
| macromolecules | 1037 | 1037 | 1037 |
| ligands | 21 | 17 | 18 |
| Protein residues | 125 | 125 | 125 |
| RMS(bonds) | 0.006 | 0.007 | 0.006 |
| RMS(angles) | 0.80 | 0.95 | 0.78 |
| Ramachandran favored (%) | 99 | 99 | 99 |
| Ramachandran allowed (%) | 0.82 | 0.82 | 0.82 |
| Ramachandran outliers (%) | 0 | 0 | 0 |
| Rotamer outliers (%) | 0.85 | 0 | 0 |
| Clashscore | 2.86 | 4.78 | 1.91 |
| Average B-factor | 20.88 | 34.82 | 22.59 |
| macromolecules | 19.51 | 34.63 | 21.12 |
| ligands | 25.82 | 32.15 | 30.16 |
| solvent | 30.09 | 37.83 | 31.93 |

Table S2. Full list of over-represented KEGG pathways after the treatment of compound BDF-1253.

| Genes in Category | Percent in the Observed List | Percent in the Genome | P-value | KEGG Pathway Name |
| --- | --- | --- | --- | --- |
| 52 | 0.060 | 0.021 | 1.19E-13 | Cell cycle |
| 20 | 0.023 | 0.007 | 1.03E-06 | Proteasome |
| 23 | 0.027 | 0.012 | 6.23E-05 | p53 signaling pathway |
| 32 | 0.037 | 0.019 | 1.11E-04 | Oocyte meiosis |
| 34 | 0.039 | 0.022 | 2.75E-04 | Spliceosome |
| 18 | 0.021 | 0.010 | 7.75E-04 | Pathogenic Escherichia coli infection |
| 39 | 0.045 | 0.028 | 1.49E-03 | Protein processing in endoplasmic reticulum |
| 16 | 0.018 | 0.009 | 1.58E-03 | Glutathione metabolism |
| 8 | 0.009 | 0.003 | 3.65E-03 | Steroid biosynthesis |
| 16 | 0.018 | 0.010 | 8.13E-03 | NOD-like receptor signaling pathway |
| 30 | 0.035 | 0.023 | 1.24E-02 | Ubiquitin mediated proteolysis |
| 63 | 0.073 | 0.056 | 1.25E-02 | Pathways in cancer |
| 18 | 0.021 | 0.012 | 1.73E-02 | Adherens junction |
| 10 | 0.012 | 0.006 | 1.73E-02 | Base excision repair |
| 28 | 0.032 | 0.022 | 2.08E-02 | Axon guidance |
| 17 | 0.020 | 0.012 | 2.35E-02 | Pancreatic cancer |
| 6 | 0.007 | 0.003 | 2.96E-02 | Selenocompound metabolism |
| 10 | 0.012 | 0.006 | 3.16E-02 | DNA replication |
| 31 | 0.036 | 0.026 | 3.24E-02 | RNA transport |
| 11 | 0.013 | 0.007 | 3.74E-02 | Bladder cancer |
| 15 | 0.017 | 0.011 | 3.78E-02 | Aminoacyl-tRNA biosynthesis |
| 19 | 0.022 | 0.015 | 4.32E-02 | Progesterone-mediated oocyte maturation |
| 18 | 0.021 | 0.014 | 6.24E-02 | TGF-beta signaling pathway |
| 8 | 0.009 | 0.005 | 6.43E-02 | Mucin type O-Glycan biosynthesis |
| 8 | 0.009 | 0.005 | 8.88E-02 | Glycine |
| 12 | 0.014 | 0.009 | 9.19E-02 | Arginine and proline metabolism |
| 6 | 0.007 | 0.004 | 9.39E-02 | beta-Alanine metabolism |

Table S3. Full list of realtime PCR primers

| **Gene** | **Forward** | **Reverse** |
| --- | --- | --- |
| c-Myc | GTGCTCCATGAGGAGACACC | GCACCTCTTGAGGACCAGTG |
| Bcl2 | GTTTCAAATCAGCTATAACTGGAG | TAATATCAGTCTACTTCCTCTGTG |
| CDK6 | TCTAACCTCAGTGGTCGTCAC | TTCTCCTGGGAGTCCAATCAC |
| BUB1 | TGGGAAAGATACATACAGTGGGT | AGGGGATGACAGGGTTCCAAT |
| CCNB1 | AATAAGGCGAAGATCAACATGGC | TTTGTTACCAATGTCCCCAAGAG |
| CDK1 | AAACTACAGGTCAAGTGGTAGCC | TCCTGCATAAGCACATCCTGA |
| PTTG1 | GCAAACCCCTCCAACCAAA | ATAGCCCGCACTCACCTTTTT |
| RBL1 | AGTAACAGGAACAACAGGACATAAAG | GAATGAGCAGTAAGTGATACAGGAC |
| YWHAH | TTGGTAGGTAGAGAGGTGGGCATA | AAGACTGTACGACACGGTGTTTTTATT |
| GADD45A | AGCAGAAGACCGAAAGGATG | GACTCCGAGCCTTGCTGA |
| DDB2 | GGGAAGATGATGTGTCAGC | CATGTGTGGGCTCCAAG |
| CDC20 | GACCACTCCTAGCAAACCTGG | GGGCGTCTGGCTGTTTTCA |
| β2-microglobulin | AAGTTGACTTACTGAAGAATGGAG | ATGCTGCTTACATGTCTCGATC |

**Supplementary figures:**


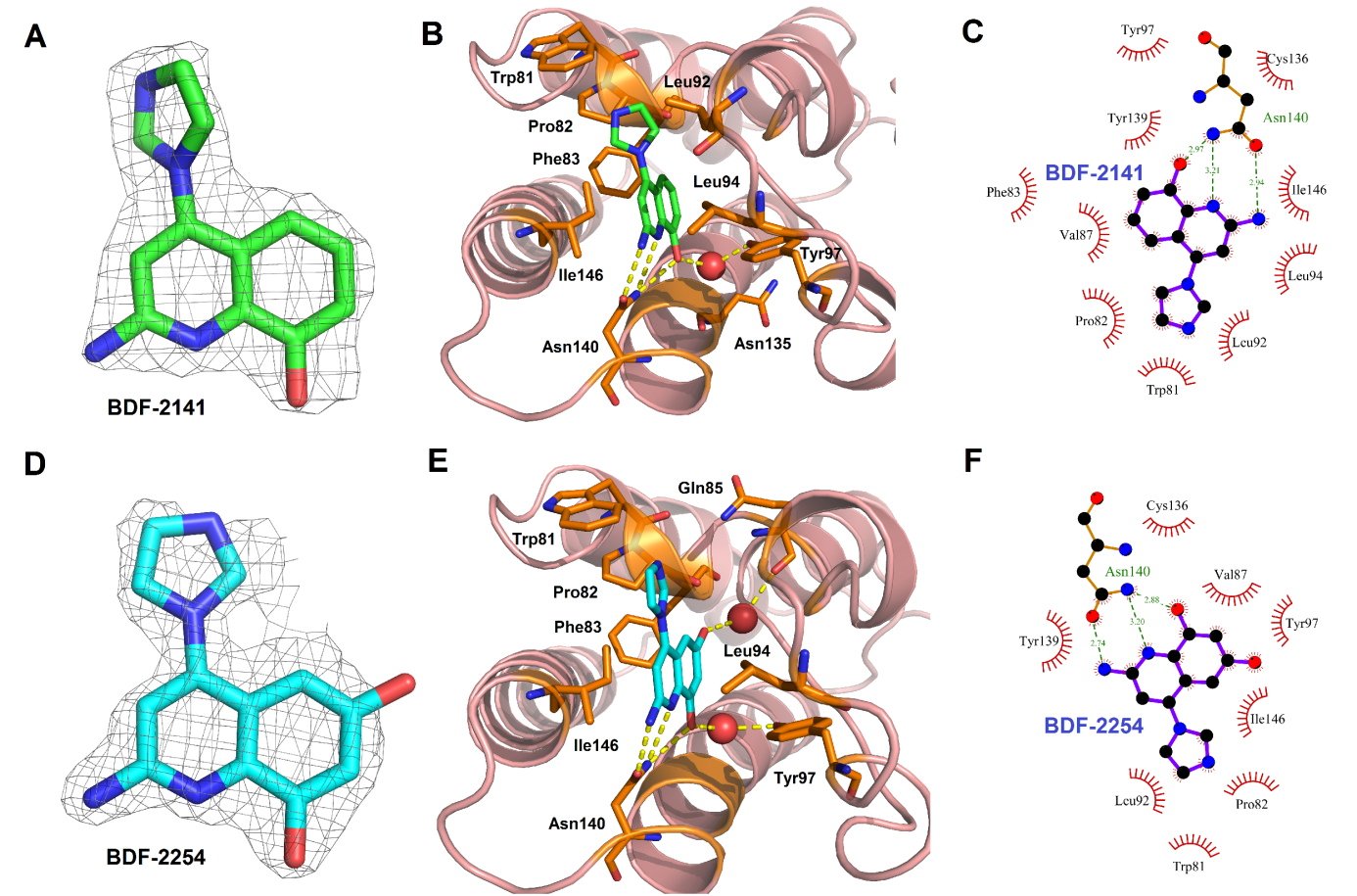


Figure S1. Complex crystal structures of compound BDF-2141 and BDF-2254, with BRD4-BD1.

(A) The 2Fo-Fc electron density map of compound BDF-2141, in its complex crystal structure with BRD4-BD1 (PDB code: 5Z5T). The density contour level was set to 1.0 sigma.

(B) Compound BDF-2141 in complex with BRD4-BD1, direct and water-bridged hydrogen bonds were formed between the compound and surrounding residues.

(C) Hydrophobic contacts of compound BDF-2141 with surrounding residues within the binding pocket.

(D) The 2Fo-Fc electron density map of compound BDF-2254, in its complex crystal structure with BRD4-BD1 (PDB code: 5Z5U). The density contour level was set to 1.0 sigma.

(E) Compound BDF-2254 in complex with BRD4-BD1, direct and water-bridged hydrogen bonds were formed between the compound and surrounding residues.

(F) Hydrophobic contacts of compound BDF-2254 with surrounding residues within the binding pocket.


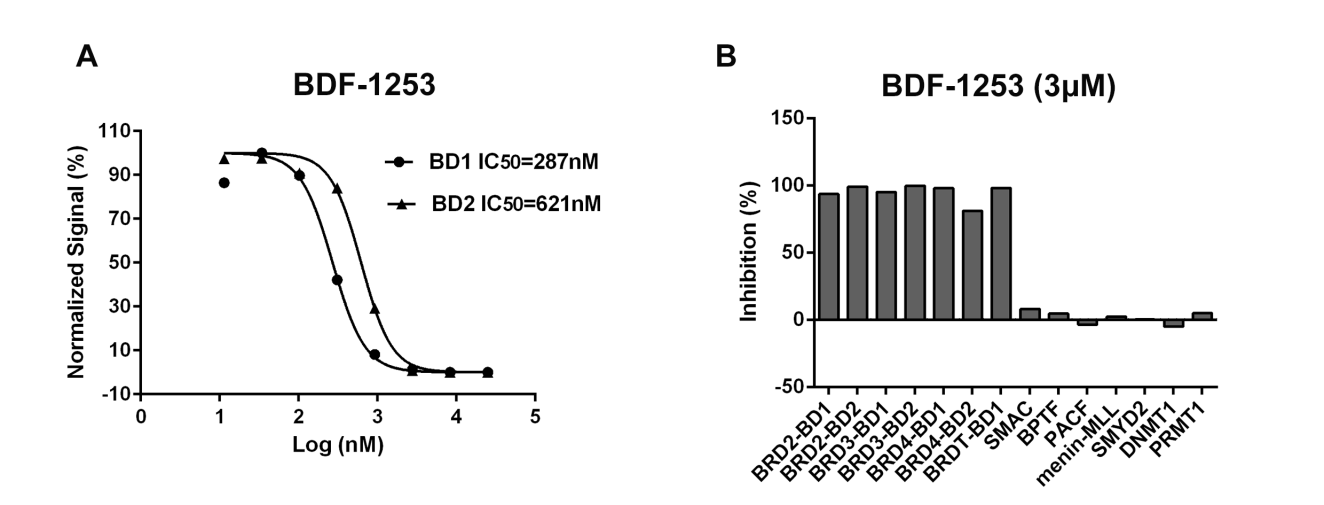


Figure S2. Determination of the selectivity of BDF-1253 in biochemical assays.

1. IC_50_ determination of BDF-1253 against BRD4-BD1 and BRD4-BD2 using Alpha Screen method.
2. Determination of inhibition against BET family members, bromodomain proteins out of the BET family as well as several epi-enzymes by BDF-1253 at 3 μM.


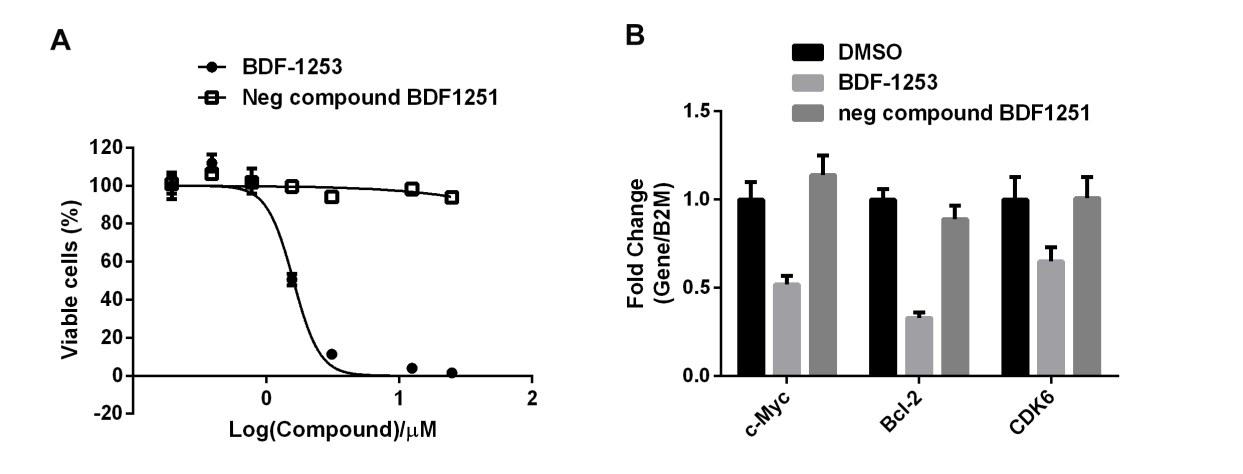


Figure S3. Comparison of the effects of BDF-1253 and negative compound BDF-1251 on cell proliferation as well as on BET target gene transcription.

(A) Compound BDF-1253 inhibited the proliferation of RCC 786-o cells while negative compound BDF-1251 showed minimal effects. Error bar indicated standard deviations among 3 technical replicates.

(B) Compound BDF-1253 inhibited expression of BET target gene c-Myc, Bcl-2 and CDK6, while negative compound BDF-1251 showed minimal effects. Gene specific data were normalized to β2-microglobulin expression and shown as average expression relative to DMSO control. Error bars indicated standard deviations among 3 technical replicates per experiment.

**Supplementary methods:**

Synthesis, purification, and characterization of the compounds

BDF-2141

Compound a (1.2 g, 1.0 eq) and imidazole (1.2 eq) were dissolved in DMF (10 mL), and sodium hydrogen (1.2 eq) was added in portions under ice-water bath. After the addition was completed, the mixture was slowly warmed to room temperature overnight until complete. The mixture was quenched with water, extracted with EA twice, and the organic phase was washed with saturated brine 4 times, dried, concentrated, and purified by column chromatography to give compound b (674 mg).

Compound b (674 mg, 1.0 eq) and p-methoxybenzylamine (4.0 eq) were dissolved in DMSO (5 mL) and heated to 95°C overnight. TLC detected the reaction was complete. Cool, quench with water, extract twice with EA, combine EA phases, wash with saturated brine 3 times, dry, concentrate and purify by column chromatography to give compound c (650 mg).

Compound c (500 mg, 1.0 eq) was dissolved in 33% HBr in HOAc solution, heated at 95° C., After the reaction was completed, EA was extracted with saturated sodium bicarbonate, dried and evaporated to give a crude product. Chromatography gave compound d (Compound BDF-2141) (330 mg).

1H NMR (400 MHz, DMSO-d6) δ 9.45 (s, 1H), 8.06 (s, 1H), 7.63 (s, 1H), 7.21 (s, 1H), 6.89 (d, J = 10.6 Hz, 2H), 6.62 (s, 2H), 6.52 (d, J = 10.0 Hz, 1H).

BDF-2246

Compound a (1.2 g, 1.0 eq) and imidazole (1.2 eq) were dissolved in DMF (10 mL), and sodium hydrogen (1.2 eq) was added in portions under ice-water bath. After the addition was completed, the mixture was slowly warmed to room temperature overnight until complete. The mixture was quenched with water, extracted with EA twice, and the organic phase was washed with saturated brine 4 times, dried, concentrated, and purified by column chromatography to give compound b (674 mg).

Compound b (674 mg, 1.0 eq) and p-methoxybenzylamine (4.0 eq) were dissolved in DMSO (5 mL) and heated to 95 °C overnight. TLC detected the reaction was complete. Cool, quench with water, extract twice with EA, combine EA phases, wash with saturated brine 3 times, dry, concentrate and purify by column chromatography to give compound c (650 mg).

Compound c (500 mg, 1.0 eq) was dissolved in 33% HBr in HOAc solution, heated at 95° C., After the reaction was completed, EA was extracted with saturated sodium bicarbonate, dried and evaporated to give a crude product. Chromatography gave compound d (Compound BDF-2141) (330 mg).

1H NMR (400 MHz, DMSO-d6) δ 8.14 (d, J = 15.0 Hz, 1H), 7.44 (dd, J = 15.0, 0.6 Hz, 1H), 7.27 (d, J = 15.0 Hz, 1H), 7.16 (d, J = 10.6 Hz, 2H), 7.07 (s, 1H), 6.50 (d, J = 15.0 Hz, 1H), 1.89 (s, 2H).

BDF-2286

Imidazole (0.36 g, 3 eq) was dissolved in anhydrous DMF (10 ml) and 60% NaH (2.5 eq) was added and stirred at room temperature for 5 minutes. Then, compound a was added and stirred at room temperature for 1 hour. The reaction solution was slowly poured into ice water and extracted with EA. The organic phase was washed with water four times, washed with brine, dried, and concentrated to give a crude product b which was directly used in the next step.

The crude product b was dissolved in ethanol (10 ml)/water (10 ml) and K2CO3 (4 eq) was weighed and the reaction was refluxed overnight at 80°C. The reaction solution was cooled to room temperature, poured into ice water, extracted with DCM, washed with water, washed with brine, dried, and concentrated. The crude product was purified by column chromatography to give 250 mg of the desired product c.

Compound c (250 mg) was dissolved in saturated HCl(g)/MeOH (10 ml) and stirred at room temperature for 3 h. The precipitated solid was filtered, the residue was dissolved in 10 mL of ammonia methanol solution, stirred for 30 minutes, and the solvent was evaporated to dryness to obtain a solid which was washed with water/methanol to give the target compound d (Compound BDF-2286) (92 mg).

1HNMR (400 MHz, CD3OD) δ 9.16(s, 1H), 8.17(s, 1H), 7.52-7.80(m, 2H), 7.40-7.49(m, 2H), 7.25-7.27(d, J=8.0Hz, 1H), 6.04(s, 2H).

BDF-2287

 1H-1,2,4-triazole (0.36 g, 3 eq) was dissolved in anhydrous DMF (10 ml) and 60% NaH (2.5 eq) was added and stirred at room temperature for 5 minutes. Then, compound a was added and stirred at room temperature for 1 hour. The reaction solution was slowly poured into ice water and extracted with EA. The organic phase was washed with water four times, washed with brine, dried, and concentrated to give a crude product b which was directly used in the next step.

The crude product b was dissolved in ethanol (10 ml)/water (10 ml) and K2CO3 (4 eq) was weighed and the reaction was refluxed overnight at 80°C. The reaction solution was cooled to room temperature, poured into ice water, extracted with DCM, washed with water, washed with brine, dried, and concentrated. The crude product was purified by column chromatography to give 250 mg of the desired product c.

Compound c (250 mg) was dissolved in saturated HCl(g)/MeOH (10 ml) and stirred at room temperature for 3 h. The precipitated solid was filtered, the residue was dissolved in 10 mL of ammonia methanol solution, stirred for 30 minutes, and the solvent was evaporated to dryness to obtain a solid which was washed with water/methanol to give the target compound d (Compound BDF-2286) (92 mg).

1HNMR (400 MHz, DMSO-d6) δ12.82(s, 1H), 11.44(br, 1H), 8.78(s, 1H), 8.17(s, 1H), 7.49-7.51(d, J=8.0Hz, 1H), 7.29-7.38(m, 2H), 6.38(s, 1H), 5.98(s, 2H).

BDF-2254

Compound a (1.2 g, 1.0 eq) and imidazole (1.2 eq) were dissolved in DMF (10 mL), and sodium hydrogen (1.2 eq) was added in portions under ice-water bath. After the addition was completed, the mixture was slowly warmed to room temperature overnight until complete. The mixture was quenched with water, extracted with EA twice, and the organic phase was washed with saturated brine 4 times, dried, concentrated, and purified by column chromatography to give compound b (674 mg).

Compound b (674 mg, 1.0 eq) and p-methoxybenzylamine (4.0 eq) were dissolved in DMSO (5 mL) and heated to 95 °C overnight. TLC detected the reaction was complete. Cool, quench with water, extract twice with EA, combine EA phases, wash with saturated brine 3 times, dry, concentrate and purify by column chromatography to give compound c (650 mg).

Compound c (500 mg, 1.0 eq) was dissolved in 33% HBr in HOAc solution, heated at 95° C, After the reaction was completed, EA was extracted with saturated sodium bicarbonate, dried and evaporated to give a crude product. Chromatography gave compound d (Compound BDF-2254) (330 mg).

1H NMR (400 MHz, DMSO-d6 ) δ 8.00 (d, J = 8.1 Hz, 1H), 7.84 (d, J = 8.1 Hz, 1H), 7.27 (dd, J = 8.0, 0.6 Hz, 1H), 7.15 (s, 1H), 6.72 (d, J = 8.1 Hz, 1H), 6.64 (s, 1H), 4.91 (s, 1H), 1.98 (s, 2H).

BDF-2250

Compound a (1.2 g, 1.0 eq) and imidazole (1.2 eq) were dissolved in DMF (10 mL), and sodium hydrogen (1.2 eq) was added in portions under ice-water bath. After the addition was completed, the mixture was slowly warmed to room temperature overnight until complete. The mixture was quenched with water, extracted with EA twice, and the organic phase was washed with saturated brine 4 times, dried, concentrated, and purified by column chromatography to give compound b (674 mg).

Compound b (674 mg, 1.0 eq) and p-methoxybenzylamine (4.0 eq) were dissolved in DMSO (5 mL) and heated to 95 °C overnight. TLC detected the reaction was complete. Cool, quench with water, extract twice with EA, combine EA phases, wash with saturated brine 3 times, dry, concentrate and purify by column chromatography to give compound c (650 mg).

Compound c (500 mg, 1.0 eq) was dissolved in 33% HBr in HOAc solution, heated at 95° C, After the reaction was completed, EA was extracted with saturated sodium bicarbonate, dried and evaporated to give a crude product. Chromatography gave compound d (Compound BDF-2250) (330 mg).

1H NMR (400 MHz, DMSO-d6) δ 12.74 (s, 1H), 9.97 (s, 1H), 9.31 (d, J = 41.1 Hz, 2H), 8.14 (d, J = 41.3 Hz, 1H), 8.08 (t, J = 1.5 Hz, 1H), 7.86 (s, 1H), 6.92 (d, J = 3.0 Hz, 2H), 6.88 (s, 1H).

BDF-2298

Imidazole (0.36 g, 3 eq) was dissolved in anhydrous DMF (10 ml) and 60% NaH (2.5 eq) was added and stirred at room temperature for 5 minutes. Then, compound a was added and stirred at room temperature for 1 hour. The reaction solution was slowly poured into ice water and extracted with EA. The organic phase was washed with water four times, washed with brine, dried, and concentrated to give a crude product b which was directly used in the next step.

The crude product b was dissolved in ethanol (10 ml)/water (10 ml) and K2CO3 (4 eq) was weighed and the reaction was refluxed overnight at 80°C. The reaction solution was cooled to room temperature, poured into ice water, extracted with DCM, washed with water, washed with brine, dried, and concentrated. The crude product was purified by column chromatography to give 250 mg of the desired product c.

Compound c (250 mg) was dissolved in saturated HCl(g)/MeOH (10 ml) and stirred at room temperature for 3 h. The precipitated solid was filtered, the residue was dissolved in 10 mL of ammonia methanol solution, stirred for 30 minutes, and the solvent was evaporated to dryness to obtain a solid which was washed with water/methanol to give the target compound d (Compound BDF-2298) (92 mg).

1HNMR (400 MHz, D2O) δ 8.23(s, 1H), 7.52(d, J = 7.2 Hz, 2H), 6.83(s, 1H), 6.77(d, J = 2.4Hz, 1H), 6.70 (br, 1H), 5.86(s, 2H).

BDF-2265

Imidazole (0.36 g, 3 eq) was dissolved in anhydrous DMF (10 ml) and 60% NaH (2.5 eq) was added and stirred at room temperature for 5 minutes. Then, compound a was added and stirred at room temperature for 1 hour. The reaction solution was slowly poured into ice water and extracted with EA. The organic phase was washed with water four times, washed with brine, dried, and concentrated to give a crude product b which was directly used in the next step.

The compound b (250 mg) was dissolved in saturated HCl(g)/MeOH (10 ml) and stirred at room temperature for 3 h. The precipitated solid was filtered, and the filter residue was dissolved in 10 mL of ammonia methanol solution, stirred for 30 minutes, and the solvent was evaporated to dryness to obtain a solid which was washed with water/methanol to give the target compound c (Compound 2265) (92 mg).

1HNMR (400 MHz, D2O) δ 8.23(s, 1H), 7.52(d, J = 7.2 Hz, 2H), 6.83(s, 1H), 6.77(d, J = 2.4Hz, 1H), 6.70 (br, 1H), 5.86(s, 2H).

BDF-2259

Compound a (145 mg, 1.0 eq) was dissolved in THF (5 mL), TBAF (1.1 eq) was added, and the reaction was heated to 50° C. for 1 h. The reaction was complete by TLC. Concentrate, and the residue was recrystallized with methanol and water and filtered to give an off-white solid b (Compound BDF-2259) (35 mg).

1H NMR (400 MHz, DMSO-d6) δ 10.81 (s, 1H), 9.84 (s, 1H), 9.79 (s, 1H), 8.17 (s, 1H), 8.05 (s, 1H), 7.61 (s, 1H), 7.22 (s, 1H), 6.75 (d, J = 2.4 Hz, 1H), 6.34 (d, J = 2.4 Hz, 1H), 2.15 (s, 3H).

BDF-2138

Compound a (1.2 g, 1.0 eq) and imidazole (1.2 eq) were dissolved in DMF (10 mL), and sodium hydrogen (1.2 eq) was added in portions under ice-water bath. After the addition was completed, the mixture was slowly warmed to room temperature overnight until complete. The mixture was quenched with water, extracted with EA twice, and the organic phase was washed with saturated brine 4 times, dried, concentrated, and purified by column chromatography to give compound b (674 mg).

Compound b (674 mg, 1.0 eq) and p-methoxybenzylamine (4.0 eq) were dissolved in DMSO (5 mL) and heated to 95°C overnight. TLC detected the reaction was complete. Cool, quench with water, extract twice with EA, combine EA phases, wash with saturated brine 3 times, dry, concentrate and purify by column chromatography to give compound c (650 mg).

Compound c (500 mg, 1.0 eq) was dissolved in 33% HBr in HOAc solution, heated at 95° C. After the reaction was completed, EA was extracted with saturated sodium bicarbonate, dried and evaporated to give a crude product. Chromatography gave compound d (Compound BDF-2138) (330 mg).

1H NMR (400 MHz, DMSO-d6) δ 8.61 (s, 1H), 7.94 (s, 1H), 7.51 (s, 1H), 7.07 (s, 1H), 6.94 – 6.75 (m, 5H).

BDF-2030

Compound a (1.2 g, 1.0 eq) and imidazole (1.2 eq) were dissolved in DMF (10 mL), and sodium hydrogen (1.2 eq) was added in portions under ice-water bath. After the addition was completed, the mixture was slowly warmed to room temperature overnight until complete. The mixture was quenched with water, extracted with EA twice, and the organic phase was washed with saturated brine 4 times, dried, concentrated, and purified by column chromatography to give compound b (674 mg).

Compound b (674 mg, 1.0 eq) and p-methoxybenzylamine (4.0 eq) were dissolved in DMSO (5 mL) and heated to 95°C overnight. TLC detected the reaction was complete. Cool, quench with water, extract twice with EA, combine EA phases, wash with saturated brine 3 times, dry, concentrate and purify by column chromatography to give compound c (650 mg).

Compound c (500 mg, 1.0 eq) was dissolved in 33% HBr in HOAc solution, heated at 95°C. After the reaction was completed, EA was extracted with saturated sodium bicarbonate, dried and evaporated to give a crude product. Chromatography gave compound d (Compound BDF-2030) (330 mg).

1H NMR (400 MHz, DMSO-d6) δ 9.45 (s, 1H), 8.06 (s, 1H), 7.63 (s, 1H), 7.21 (s, 1H), 6.89 (d, J = 10.6 Hz, 2H), 6.62 (s, 2H), 6.52 (d, J = 10.0 Hz, 1H).

BDF-2268

Imidazole (0.36 g, 3 eq) was dissolved in anhydrous DMF (10 ml) and 60% NaH (2.5 eq) was added and stirred at room temperature for 5 minutes. Then, compound a was added and stirred at room temperature for 1 hour. The reaction solution was slowly poured into ice water and extracted with EA. The organic phase was washed with water four times, washed with brine, dried, and concentrated to give a crude product b which was directly used in the next step.

Compound b was dissolved in ethanol (10 ml)/water (10 ml) and K2CO3 (4 eq) was weighed and the reaction was refluxed overnight at 80°C. The reaction solution was cooled to room temperature, poured into ice water, extracted with DCM, washed with water, washed with brine, dried, and concentrated. The crude product was purified by column chromatography to give 250 mg of the desired product c.

Compound c (250 mg) was dissolved in saturated HCl(g)/MeOH (10 ml) and stirred at room temperature for 3 h. The precipitated solid was filtered, the residue was dissolved in 10 mL of ammonia methanol solution, stirred for 30 minutes, and the solvent was evaporated to dryness to obtain a solid which was washed with water/methanol to give the target compound d (Compound BDF-2268) (92 mg).

1HNMR (400 MHz, CD3OD) δ 8.176(s, 1H), 7.81(s, 1H), 7.78(s, 1H), 7.27(dd, J=9.2, 2.4Hz, 1H), 7.06(dd, J=9.6, 2.0Hz, 1H), 6.61(s, 1H), 5.99 (s, 2H).

BDF-2271

Imidazole (0.36 g, 3 eq) was dissolved in anhydrous DMF (10 ml) and 60% NaH (2.5 eq) was added and stirred at room temperature for 5 minutes. Then, compound a was added and stirred at room temperature for 1 hour. The reaction solution was slowly poured into ice water and extracted with EA. The organic phase was washed with water four times, washed with brine, dried, and concentrated to give a crude product b which was directly used in the next step.

Compound b was dissolved in ethanol (10 ml)/water (10 ml) and K2CO3 (4 eq) was weighed and the reaction was refluxed overnight at 80°C. The reaction solution was cooled to room temperature, poured into ice water, extracted with DCM, washed with water, washed with brine, dried, and concentrated. The crude product was purified by column chromatography to give 250 mg of the desired product c.

Compound c (250 mg) was dissolved in saturated HCl(g)/MeOH (10 ml) and stirred at room temperature for 3 h. The precipitated solid was filtered, the residue was dissolved in 10 mL of ammonia methanol solution, stirred for 30 minutes, and the solvent was evaporated to dryness to obtain a solid which was washed with water/methanol to give the target compound d (Compound BDF-2268) (92 mg).

1HNMR (400 MHz, DMSO-d6) δ 8.75(s, 1H), 8.09(s, 1H), 7.10-7.13(m, 1H), 6.80-6.84(m, 1H), 6.40(s, 1H), 6.28(s, 1H), 5.74(s, 2H).

BDF-2143

Compound a (1.2 g, 1.0 eq) and imidazole (1.2 eq) were dissolved in DMF (10 mL), and sodium hydrogen (1.2 eq) was added in portions under ice-water bath. After the addition was completed, the mixture was slowly warmed to room temperature overnight until complete. The mixture was quenched with water, extracted with EA twice, and the organic phase was washed with saturated brine 4 times, dried, concentrated, and purified by column chromatography to give compound b (674 mg).

Compound b (674 mg, 1.0 eq) and p-methoxybenzylamine (4.0 eq) were dissolved in DMSO (5 mL) and heated to 95°C overnight. TLC detected the reaction was complete. Cool, quench with water, extract twice with EA, combine EA phases, wash with saturated brine 3 times, dry, concentrate and purify by column chromatography to give compound c (650 mg).

Compound c (500 mg, 1.0 eq) was dissolved in 33% HBr in HOAc solution, heated at 95°C. After the reaction was completed, EA was extracted with saturated sodium bicarbonate, dried and evaporated to give a crude product. Chromatography gave compound d (Compound BDF-2143) (330 mg).

1H NMR (400 MHz, DMSO-d6) δ 8.07 (s, 1H), 7.64 (s, 1H), 7.22 (s, 1H), 7.11 (d, J = 7.9 Hz, 1H), 6.90 (d, J = 7.9 Hz, 1H), 6.85 (s, 1H), 6.80 (s, 2H).

BDF-2243

Compound a (145 mg, 1.0 eq) was dissolved in THF (5 mL), TBAF (1.1 eq) was added, and the reaction was heated to 50°C for 1 h. The reaction was complete by TLC. Concentrate, and the residue was recrystallized with methanol and water and filtered to give an off-white solid b (Compound BDF-2243) (35 mg).

1H NMR (400 MHz, DMSO-d6) δ 10.92 (s, 1H), 8.30 (s, 1H), 8.14 (s, 1H), 7.70 (s, 1H), 7.64 (d, J = 9.1 Hz, 1H), 7.26 (s, 1H), 7.08 (d, J = 9.1 Hz, 1H), 2.21 (s, 3H).

BDF-1031

Compound a (145 mg, 1.0 eq) was dissolved in THF (5 mL), TBAF (1.1 eq) was added, and the reaction was heated to 50°C for 1 h. The reaction was complete by TLC. Concentrate, and the residue was recrystallized with methanol and water and filtered to give an off-white solid b (Compound BDF-1031) (35 mg).

1H NMR (400 MHz, DMSO-d6) δ 11.00 (s, 1H), 9.75 (s, 1H), 8.30 (s, 1H), 8.10 (s, 1H), 7.67 (s, 1H), 7.37 (d, J = 6.3 Hz, 1H), 7.25 (s, 1H), 7.17 (d, J = 10.4 Hz, 1H), 7.06 (s, 1H), 2.18 (s, 3H).

BDF-1253

Imidazole (0.36 g, 3 eq) was dissolved in anhydrous DMF (10 ml) and 60% NaH (2.5 eq) was added and stirred at room temperature for 5 minutes. Then, raw material a was added and stirred at room temperature for 1 hour. The reaction solution was slowly poured into ice water and extracted with EA. The organic phase was washed with water four times, washed with brine, dried, and concentrated to give a crude product b which was directly used in the next step.

Compound b (250 mg) was dissolved in saturated HCl(g)/MeOH (10 ml) and stirred at room temperature for 3 h. The precipitated solid was filtered, and the filter residue was dissolved in 10 mL of ammonia methanol solution, stirred for 30 minutes, and the solvent was evaporated to dryness to obtain a solid which was washed with water/methanol to give the target compound c (Compound BDF-1253) (92 mg).

1HNMR(400 MHz, CD3OD)δ10.64 (s, 1H), 9.32-9.36(br, 1H), 7.76（s, 1H），7.73（s, 1H）, 7.47-7.49(d, J = 8.0 Hz, 1H), 7.31-7.35(t, J = 8.0Hz, 1H), 7.17(s, 1H), 7.07-7.09(d, J = 8.0 Hz, 1H), 6.93(s, 1H)， 5.69 (s, 2H), 2.01(s, 3H).

BDF-1252

1H-1,2,4-triazole (0.36 g, 3 eq) was dissolved in anhydrous DMF (10 ml) and 60% NaH (2.5 eq) was added and stirred at room temperature for 5 minutes. Then, compound a was added and stirred at room temperature for 1 hour. The reaction solution was slowly poured into ice water and extracted with EA. The organic phase was washed with water four times, washed with brine, dried, and concentrated to give a crude product b which was directly used in the next step.

Compound b (250 mg) was dissolved in saturated HCl(g)/MeOH (10 ml) and stirred at room temperature for 3 h. The precipitated solid was filtered, and the filter residue was dissolved in 10 mL of ammonia methanol solution, stirred for 30 minutes, and the solvent was evaporated to dryness to obtain a solid which was washed with water/methanol to give the target compound c (Compound BDF-1252) (92 mg).

1HNMR (400 MHz, DMSO-d6) δ 8.56(s, 1H), 8.35(s, 1H), 8.74(s, 1H), 8.05(s, 1H), 7.84(s, 1H), 7.53(d, J = 7.6 Hz, 1H), 7.35(t, J = 8.0 Hz , 1H), 7.10(d, J = 6.8 Hz, 1H), 5.93(s, 2H), 2.11(s, 2H).

BDF-1251

1H-1,2,4-triazole (0.36 g, 3 eq) was dissolved in anhydrous DMF (10 ml) and 60% NaH (2.5 eq) was added and stirred at room temperature for 5 minutes. Then, compound a was added and stirred at room temperature for 1 hour. The reaction solution was slowly poured into ice water and extracted with EA. The organic phase was washed with water four times, washed with brine, dried, and concentrated to give a crude product b (compound BDF-1251)

1HNMR (400 MHz, DMSO-d6) δ9.03 (s, 1H), 8.74 (s, 1H), 8.04(s, 1H), 7.93(s, 1H), 7.65(d, J = 8.8 Hz, 1H), 7.45(t, J = 7.6 Hz , 1H), 7.20(d, J = 7.6 Hz, 1H), 5.94(s, 2H), 3.94(s, 3H), 2.09 (s, 2H).

BDF-1005

Compound a (237 mg, 1 eq.), 2-aminoimidazole (100 mg, 2 eq.), Pd2(dba)3 (50 mg, 0.1 eq.), Xphos (51 mg, 0.2 eq.) and potassium carbonate (240 mg, 3 eq.) were dissolved in tert-butanol (10 mL) and refluxed at 90° C. for 7 h. The reaction was monitored by TLC. The solvent was evaporated to dryness to give the target product b (147 mg).

Compound b (116 mg, 1 eq.) and tetrabutylammonium fluoride (70 mg, 1 eq.) in THF (10 mL), stirred at 50° C. for 4 h, complete reaction, pre-HPLC separation The target product c (compound BDF-1005 ) (20 mg) was obtained.

1H NMR (400 MHz, CD3OD) δ 8.56 (s, 1H), 7.90 (s, 1H), 7.53 (d, J = 30.0 Hz, 2H), 7.29 (d, J = 15.0 Hz, 2H), 2.32 (s, 3H).

BDF-1206

Compound a (237 mg, 1 eq.), 2-aminoimidazole (100 mg, 2 eq.), Pd2(dba)3 (50 mg, 0.1 eq.), Xphos (51 mg, 0.2 eq.) and potassium carbonate (240 mg, 3 eq.) were dissolved in tert-butanol (10 mL) and refluxed at 90°C for 7 h. The reaction was monitored by TLC. The solvent was evaporated to dryness to give the target product b (147 mg).

Compound b (116 mg, 1 eq.) and tetrabutylammonium fluoride (70 mg, 1 eq.) in THF (10 mL) were stirred at 50°C for 4 h and the reaction was complete. Pre-HPLC separation of the desired product c ( Compound BDF-1206) (20 mg).

1H NMR (400 MHz, DMSO-d6) δ 8.45 (s, 1H), 8.29 (s, 1H), 8.96 (s, 2H), 7.82 (s, 2H), 7.72 (d, J =8.4 Hz, 1H), 7.27 (t, J = 8.0 Hz, 1H), 7.06 (d, J = 7.5 Hz, 1H), 2.09 (s, 3H).

BDF-2031

Compound a (145 mg, 1.0 eq) was dissolved in THF (5 mL), TBAF (1.1 eq) was added, and the reaction was heated to 50°C for 1 h. The reaction was complete by TLC. Concentrate, and the residue was recrystallized with methanol and water and filtered to give an off-white solid b (Compound BDF-2031) (35 mg).

1H NMR (400 MHz, MeOD) δ 9.27 (s, 1H), 8.48 (s, 1H), 8.00 (s, 1H), 7.80 (s, 1H), 7.10 (s, 1H), 6.87 (s, 1H), 2.43 (s, 3H), 2.26 (s, 3H).

BDF-2242

Compound a (145 mg, 1.0 eq) was dissolved in THF (5 mL), TBAF (1.1 eq) was added, and the reaction was heated to 50° C. for 1 h. The reaction was complete by TLC. Concentrate, and the residue was recrystallized with methanol and water and filtered to give an off-white solid b (Compound BDF-2242) (35 mg).

1H NMR (400 MHz, DMSO-d6) δ 11.02 (s, 1H), 8.32 (s, 1H), 8.12 (s, 1H), 7.68 (s, 1H), 7.25 (s, 1H), 7.03 (dd, J = 10.4, 2.4 Hz, 1H), 6.69 (dd, J = 9.8, 2.4 Hz, 1H), 2.18 (s, 3H).

BDF-2260

Imidazole (0.36 g, 3 eq) was dissolved in anhydrous DMF (10 ml) and 60% NaH (2.5 eq) was added and stirred at room temperature for 5 minutes. Then, compound a was added and stirred at room temperature for 1 hour. The reaction solution was slowly poured into ice water and extracted with EA. The organic phase was washed with water four times, washed with brine, dried, and concentrated to give a crude product b.

The compound b (250 mg) was dissolved in saturated HCl(g)/MeOH (10 ml) and stirred at room temperature for 3 h. The precipitated solid was filtered, the residue was dissolved in 10 mL of ammonia methanol solution, stirred for 30 minutes, and the solvent was evaporated to dryness to obtain a solid which was washed with water/methanol to give the target compound c (Compound BDF-2261) (92 mg).

1HNMR (400 MHz,CD3OD) δ7.78（s, 1H）, 7.75(br , 1H)，7.19-7.24(br, 2H), 6.90-6.96 (br, 2H), 5.65（s, 2H）, 2.10 (s, 3H).

BDF-2261

Imidazole (0.36 g, 3 eq) was dissolved in anhydrous DMF (10 ml) and 60% NaH (2.5 eq) was added and stirred at room temperature for 5 minutes. Then, compound a was added and stirred at room temperature for 1 hour. The reaction solution was slowly poured into ice water and extracted with EA. The organic phase was washed with water four times, washed with brine, dried, and concentrated to give a crude product b.

The compound b (250 mg) was dissolved in saturated HCl(g)/MeOH (10 ml) and stirred at room temperature for 3 h. The precipitated solid was filtered, the residue was dissolved in 10 mL of ammonia methanol solution, stirred for 30 minutes, and the solvent was evaporated to dryness to obtain a solid which was washed with water/methanol to give the target compound c (Compound BDF-2261) (92 mg).

1HNMR (400 MHz, DMSO-d6) δ 10.69(s , 1H), 10.16(s , 1H), 8.76(s , 1H), 8.06 (s, 1H), 7.89（br, 1H）, 7.32-7.35(m, 1H), 6.96-6.99(m, 1H), 5.88 (s, 2H), 2.11 (s, 3H).
